# Causes of Variations in Sediment Yield in the Jinghe River Basin, China

**DOI:** 10.1038/s41598-020-74980-3

**Published:** 2020-10-22

**Authors:** Jinliang Zhang, Yizi Shang, Jinyong Liu, Jian Fu, Shitao Wei, Liang Tong

**Affiliations:** 1 Yellow River Engineering Consulting Co., Ltd, Zhengzhou, 450003 China; 2 State Key Laboratory of Simulation and Regulation of Water Cycles in River Basins, China Institute of Water Resources and Hydropower Research, Beijing, China

**Keywords:** Hydrology, Forestry, Environmental impact

## Abstract

The Jinghe River remains the major sediment source of the Yellow River in China; however, sediment discharge in the Jinghe River has reduced significantly since the 1950s. The objective of this study is to identify the causes of sediment yield variations in the Jinghe River Basin based on soil and water conservation methods and rainfall analyses. The results revealed that soil and water conservation projects were responsible for half of the total sediment reduction; sediment retention due to reservoirs and water diversion projects was responsible for 1.3% of the total reduction. Moreover, the Jinghe River Basin has negligible opportunity to improve its vegetation cover (currently 55% of the basin is covered with lawns and trees), and silt-arrester dams play a smaller role in reducing sediment significantly before they are entirely full. Therefore, new large-scale sediment trapping projects must be implemented across the Jinghe River Basin, where heavy rainfall events are likely to substantially increase in the future, leading to higher sediment discharge.

## Introduction

Climate, underlying surfaces, runoff, and sediment discharge constitute the hydrological system in a river basin. Over the past few decades, the hydrological systems of many river basins worldwide have experienced significant changes due to global warming and human activity^1–3^. By changing atmospheric circulation, warming reshapes the temporal and spatial distribution of precipitation in river basins, which, in turn, affects runoff and sediment discharge. Meanwhile, changes to the surface of the earth by human activity have altered the runoff and sediment discharge processes in river basins^4,5^. Nevertheless, runoff and sediment discharge have a complex response relationship with the climate and the underlying surfaces, which is highly nonlinear and uncertain. This renders such processes of change difficult to understand and control. In terms of specific performance, the runoff and sediment processes may differ among regions, among rivers and even among time spans along the same river^6–8^.

In North China, runoff and sediment discharge in most rivers have simultaneously decreased over the past 60 years, but the influencing factors are not entirely the same^9^. For the Yihe River, soil and water conservation (mainly referred to as water conservation works) were major contributors to recent water and sediment reductions, while rainfall had a certain effect on the reduced sediment discharge^10^. Human activity also had a notable effect on the Songhua River, affecting 52.8 and 81.8% of the runoff and sediment discharge, respectively, as compared to 47.2 and 18.2% of the changes in the runoff and sediment, respectively, due to climate change^11^. For the Liaohe River, however, rainfall was the major contributor to the decline in water and sediment, followed by human activity, including sediment trapping by water conservation works, soil and water conservation measures, section sediment stagnation, and water and sediment diversion^12^. Different from either of the rivers above, changes in rainfall affected 69.7–72.9% of the runoff in the Beiluo River, whereas human activity affected 80.5–92.1% of the sediment^13^. Similar to the Beiluo River, rainfall was the largest contributor to the variations in runoff in the Wuhua River basin, whereas human activity was the largest contributor to the variations in sediment discharge^14^. Zhang and Liu^15^ found that the reduction in sediment discharge in the Xiangjiang River from 1995–2002 was mainly attributed to the implementation of water conservation measures, whereas reductions from 2003–2011 resulted from comprehensive conservation measures, such as returning farmland to forests.

Rainfall in the basin is an important factor contributing to reduced river sediment discharge^16,17^. China suffers from severe soil and water loss, where 46% of this loss is attributable to rainfall^18^. The statistics show that a 1% variation in rainfall led to runoff and sediment load variations of 1.3% and 2%, respectively^19^. In particular, intense rainfall events can significantly increase runoff and soil erosion^20,21^^.^ Sun^22^ analysed the temporal and spatial variations in 12 extreme rainfall indicators at 10 meteorological stations in the Yanhe River Basin from 1970–2010, finding that extreme storm rainfall in the Yanhe River Basin was linearly correlated with and directly influenced by runoff and sediment discharge. Li et al.^23^ used measurement data from 1960–2012 at the Maduwang hydrometric station on the Bahe River to determine the relationship between rainfall and runoff/sediment discharge in the basin. They found that a decrease of 1 mm in the rainfall led to a decrease of 6,680 t in annual sediment discharge.

The Loess Plateau, the most severely eroded area in China, is the primary source of sediment for the Yellow River. Rainfall in this area is concentrated and largely heavy. Precipitation in the basin transports a large amount of sediment into the Yellow River^24^, rendering the Yellow River as the most sediment-laden river in the world. The annual average sediment discharge of the Yellow River can reach up to 1.6 billion t, accounting for 6% of the world's annual sediment discharge by rivers. However, due to a variety of factors, there has been a ~ 70% reduction in the runoff and sediment discharge of the Yellow River over the past 60 years. Water diversion programs, soil and water conservation measures, and rainfall were suggested as the contributors to the variations in sediment discharge^25,26^. Recent studies have further shown that changes in the underlying surface conditions, associated with engineering projects implemented by humans, are the dominant contributor to runoff and sediment reduction in the Yellow River^27–30^.

Engineering projects include soil and water conservation works, reservoirs, and water diversions. For rivers with relatively high sediment concentrations, water diversion works can remove a certain amount of sediment, thereby reducing sediment discharge in the river channel^31^. These measures can be implemented comprehensively or as a single measure. Various measures inevitably produce different effects; even the same measure across different time spans can have different effects^32–34^. Land use and surface cover conditions have evolved in the Yellow River Basin since the 1950s^26^. Especially since the 1970s, large-scale comprehensive governance of soil erosion has been conducted in the Loess Plateau. Soil and water conservation measures have been implemented, such as large-scale artificial afforestation^35^, silt-arrestor dams^36^, mountain enclosure and grazing bans^37^, and terraces^38^. In 1999, the government implemented a policy of returning farmland to forest (grassland)^39^. These studies agree that human activity has been mainly responsible for reduced sediment discharge in the Yellow River Basin^40^. Engineering projects, such as vegetation restoration, terraces, silt-arrester dams, and reservoirs, have played a significant role in soil and water conservation across the Loess Plateau^41^.

The Jinghe River, a major sediment source for the Yellow River, contributes 71% of its sediment to the Weihe River and 23% of its sediment to the Yellow River^42^. The Jinghe River Basin is the key area for sediment control with respect to the Yellow River. China has continuously implemented soil and water conservation works in the Jinghe River Basin^43^. Consequent to these conservation works over the past 60 years, the average annual sediment discharge in the Jinghe River since 2000 decreased from 153,000,000 to 97,000,000 t (61.2%), as compared with the average annual sediment discharge from 1956–1999 of 250,000,000 t^44^. However, climate change will likely modify rainfall patterns, which in turn, will increase river sediments. This necessitates the implementation of further water management strategies. This study focuses on the reasons for the decreasing sediment discharge in the Jinghe River Basin from 2000 to 2015, as compared with 1956–1999, by investigating the effects that soil and water conservation works and rainfall variation have on sediment yield.

The aim of this study is to use existing databases to estimate the reduction in sediment yield; the objective is to perform a number of calculations based on these different datasets to identify the intervention that has had the greatest effect (e.g. land use change, silt-arrestor dams, etc.). The remainder of this paper is organised as follows. Section 2 presents an overview of the study area. The location of the study area and water systems in the basin are described in Sect. 2.1, the characteristics of the sediment found in the study area are given in Sect. 2.2, and the particle size of sediment and the erosion–deposition processes in the Jinghe River are presented in Sect. 2.3. Section 3 presents the results of the calculations and analyses. Section 4 discusses the uncertainty of the calculation results. Section 5 summarises the study, and Sect. 6 presents the methods and data used in this study.

## Study area

### Overview of the Jinghe River Basin

**Figure 1:**
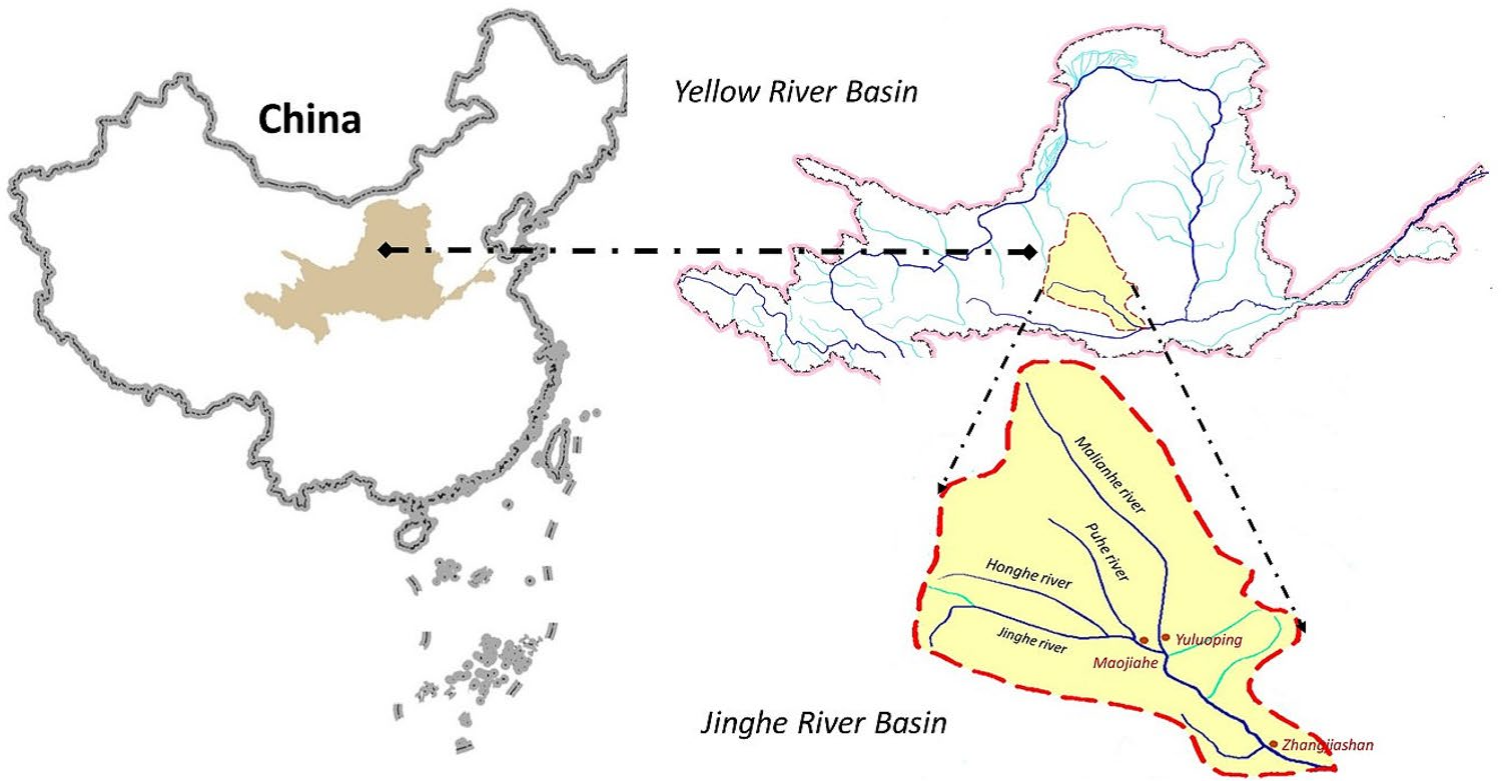
Location of the Jinghe River Basin, China, and the distribution of its water system. (The map was generated using the map-making tool on the website: https://bzdt.ch.mnr.gov.cn/).

Figure 1 depicts the study area of the Jinghe River Basin^44^. The Jinghe River is the largest tributary of the Weihe River and a Class II tributary of the Yellow River. The Jinghe River originates in Laolongtan, Jingyuan County, Ningxia Autonomous Region, on the eastern foot of the Liupan Mountain, runs through the Ningxia, Gansu, and Shaanxi provinces, and meets the Weihe River in Chenjiatan Township, Gaoling County, Shaanxi Province. The Jinghe River is 455.1 km long, with a drainage area of 45,421 km^2^. Numerous tributaries have a fan-like distribution on both banks of this river. The basin faces serious soil and water loss across 73% of its total area, resulting in high sediment concentrations in the water system. In particular, the Malianhe and Puhe River tributaries have an average annual erosion modulus (The soil erosion modulus is a unit of soil erosion intensity and a quantitative index that measures the degree of soil erosion. It is also referred to as the soil erosion rate, soil loss rate, or soil loss magnitude. This index is the amount of soil eroded and displaced per unit of area and per unit of time under the combined effects of natural forces (i.e. water, wind, gravity, freezing and thawing, etc.) and human activity, in t/(km^2^·a).) of ~ 7000–9000 t/(km^2^ a).

The Jinghe River Basin slopes from the northwest towards the southeast. The basin is surrounded by the Ziwu Mountain (with a main-peak altitude of 1,687 m) to the east, Guanshan Mountain to the south, Liupan Mountain to the west, and Yangjuan Mountain to the north. It has a vast loess plateau area at its centre. The basin can be divided by its geomorphic features into the loess hill gully area (40%), loess plateau gully area (45.8%), and rock and soil mountain forest area (14.2%). The basin has a fragmented terrain, sparse vegetation, and suffers from serious soil and water loss. The basin is in the middle reaches of the Yellow River and experiences frequent storms.

### Sediment in the Jinghe River Basin

**Figure 2:**
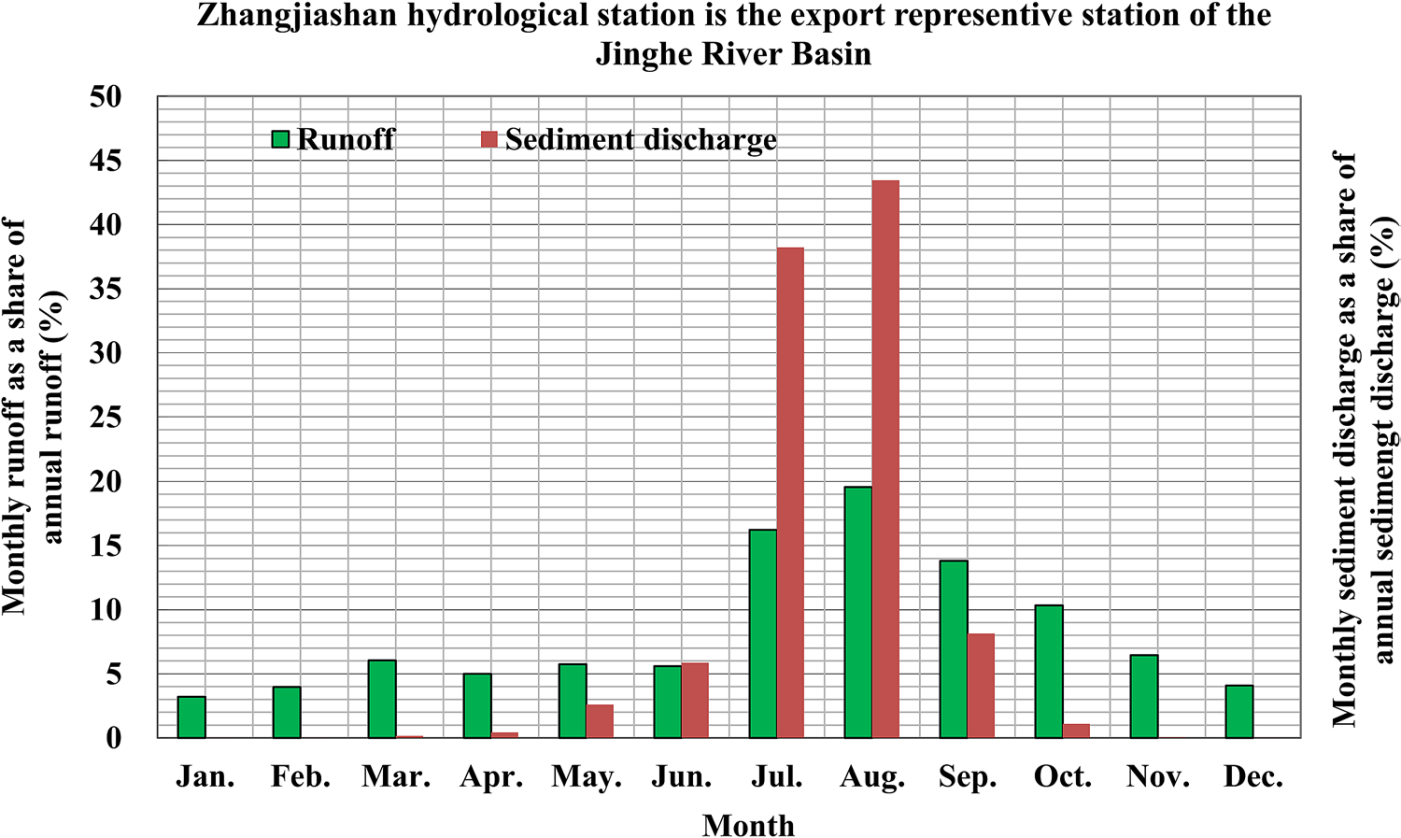
Distribution of the annual runoff and sediment discharge at the Zhangjiashan Station on the Jinghe River.

Most of the runoff in the Jinghe River Basin occurs from July to October, accounting for 61.8% of the total annual runoff. The sediment discharge from July to August accounts for 80.6% of the total annual sediment discharge (Fig. 2) (Conversion relation between 1 kg/m^3^ and 1 g/l:1 kg/m^3^ = 1 g/l.). The average sediment concentrations in July and August are the highest at 314 and 296 kg/m^3^, respectively, followed by 139 and 79 kg/m^3^ in June and September, respectively.

**Figure 3:**
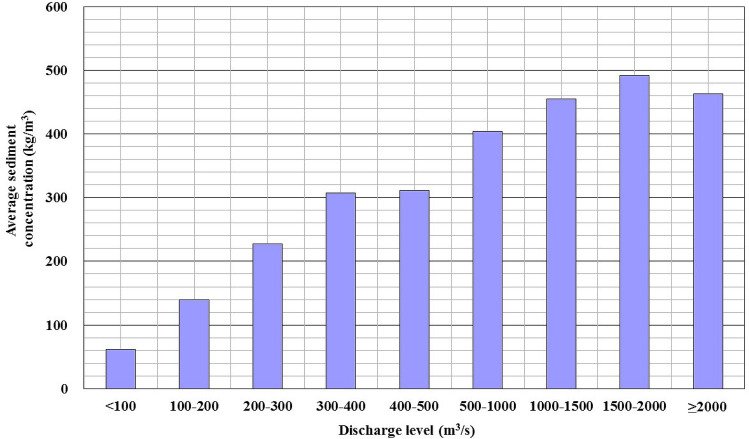
Average sediment concentration with respect to the discharge level at the Zhangjiashan Station on the Jinghe River.

Sediment concentrations in the Jinghe River Basin are high in the flood season, with a maximum measured instantaneous sediment concentration of 1,528 kg/m^3^. Figure 3 depicts the variations in the average sediment concentration and discharge levels. The average sediment concentration is 62.39 kg/m^3^ when the discharge level is lower than 100 m^3^/s. This rises rapidly to 139.66, 227.02, 307.62, and 311.17 kg/m^3^ when the discharge level reaches ~ 100–200, ~ 200–300, ~ 300–400, and ~ 400–500 m^3^/s, respectively. The sediment concentration exceeds 400 kg/m^3^ at a discharge level higher than 500 m^3^/s. Therefore, the sediment concentration increases with the discharge level and finally stabilises.

Most of the runoff in the Jinghe River Basin originates from sections upstream of the Yangjiaping Station and from the Yangjiaping and Yuluoping stations to Zhangjiashan Station, accounting for 73.1% of the runoff in the basin. The sections upstream of the Yangjiaping Station, from Yangjiaping and Yuluoping stations to Zhangjiashan Station, and upstream of Yuluoping Station comprise 42, 31, and 27% of the runoff source zones in the Jinghe River Basin, respectively.

Most sediment discharge originates from three sediment-rich tributaries, namely, the Malian, Honghe, and Puhe, accounting for 41.2% of the runoff and 76.9% of the sediment discharge. The Malian, Honghe, and Puhe river tributaries comprise 77% of the sediment source zones in the Jinghe River Basin.

Both runoff and sediment discharge in the Jinghe River Basin vary widely from one year to another. The maximum measured annual runoff was ~ 4,183,000,000 m^3^ in 1964, six times the minimum value of ~ 702,000,000 m^3^ measured in 2009. Sediment discharge varies even more from year to year; the maximum annual sediment discharge was ~ 1,171,000,000 t in 1933, 49 times the minimum value of ~ 24,000,000 t measured in 2014.

### Particle size of sediment and the erosion–deposition processes in the Jinghe River

**Figure 4:**
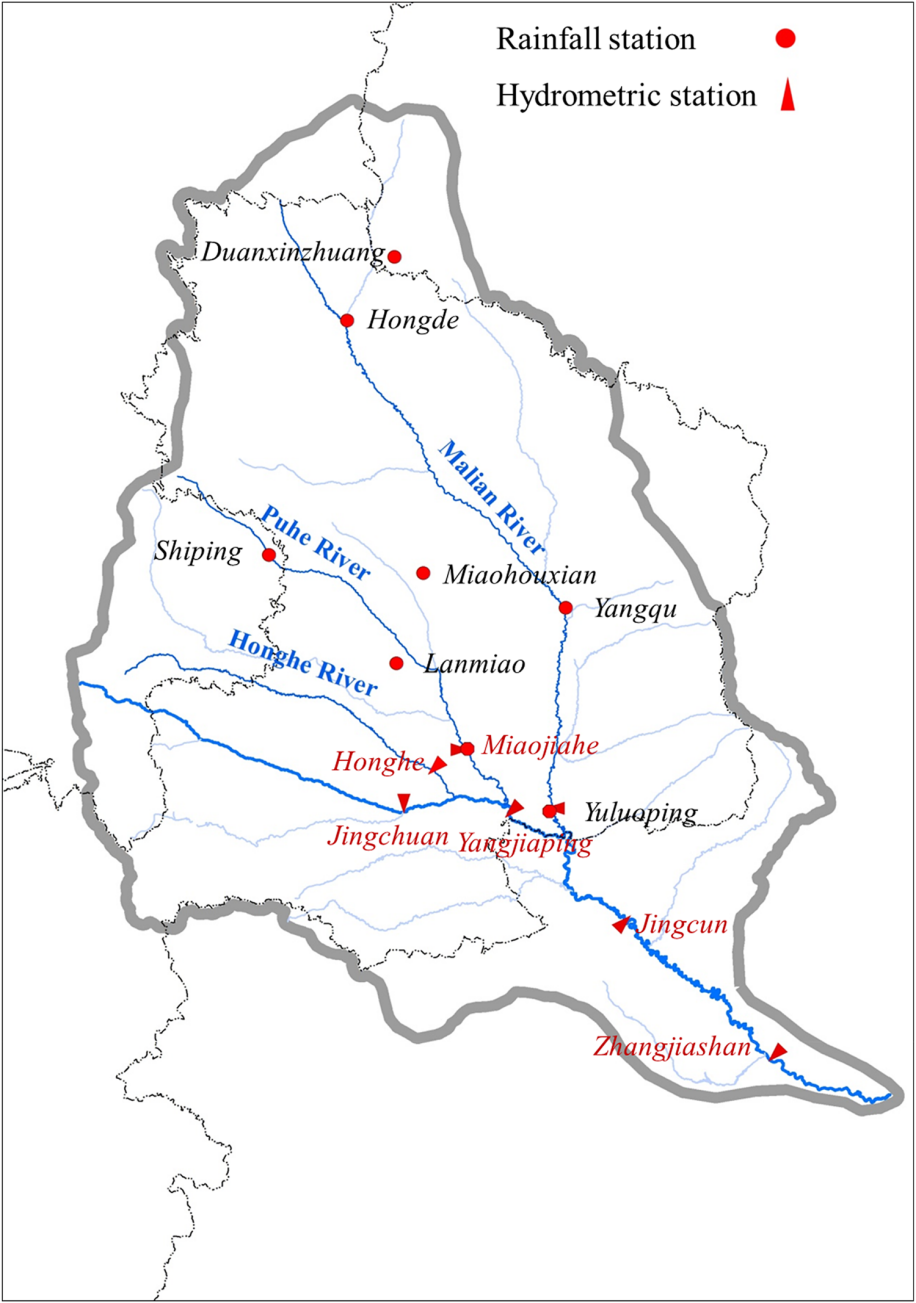
Distribution of rainfall stations in the Jinghe River Basin. (The map was generated using the map-making tool on the website: https://bzdt.ch.mnr.gov.cn/).

Table 1 lists the median particle size from various hydrological stations in the Jinghe River Basin, and the key stations in the Jinghe River Basin are depicted in Fig. 4. Here, D_50_ is used to represent the median particle size of the sediment. This factor refers to the size above or below which sediment accounts for 50% of the sample by weight. In the particle size gradation curve dynamically updated by the hydrological stations in the Jinghe River Basin, the value on the abscissa, corresponding to 50% on the ordinate, is the median particle size of the sediment.

**Table 1:** D_50_ (mm) of sediment at different stations (annual average).

Mainstream and tributaries	Station	2010	2011	2012	2013	2014	2015
Jinghe River (mainstream)	Jingchuan	0.012	0.009	0.008	0.010	0.009	0.011
	Yangjiaping	0.012	0.008	0.010	0.011	0.011	0.009
	Zhangjiashan	0.013	0.017	0.007	0.004	0.006	0.010
Malian River (tributary)	Yuluoping	0.023	0.020	0.021	0.021	0.017	0.025
Ruihe River (tributary)	Yuanjiaan	0.011	0.011	0.007	0.009	0.008	0.007

The main sediment source tributaries of the Jinghe River, such as the Malianhe River, have a large sediment particle size of ~ 0.017 to 0.027 mm. Other tributaries, such as the Puhe River, have a sediment particle size of ~ 0.006 to 0.011 mm. However, due to the influence of inflows from upstream tributaries or erosion–deposition changes in the above reaches, the median particle size of the sediment varies widely in the Jinghe River mainstream. The median particle size ranges from ~ 0.008–0.012 mm in the Jingchuan section, ~ 0.007–0.012 mm in the Yangjiaping section, and ~ 0.004–0.018 mm in the Zhangjiashan section.

By analysing the changes in the median particle size of the sediment at different hydrological station sections of the mainstream and tributaries, conclusions on the erosion and deposition processes can be obtained. As listed in Table 1, the median particle size in certain downstream sections of the Jinghe mainstream is smaller than that of the upstream sections in 2012, 2013, and 2017, indicating that there may be sedimentation in these reaches. However, in general, the Jinghe River mainstream has been in a balanced erosion–deposition state since 2010.

## Results and analysis

### Sediment concentration analysis

**Figure 5:**
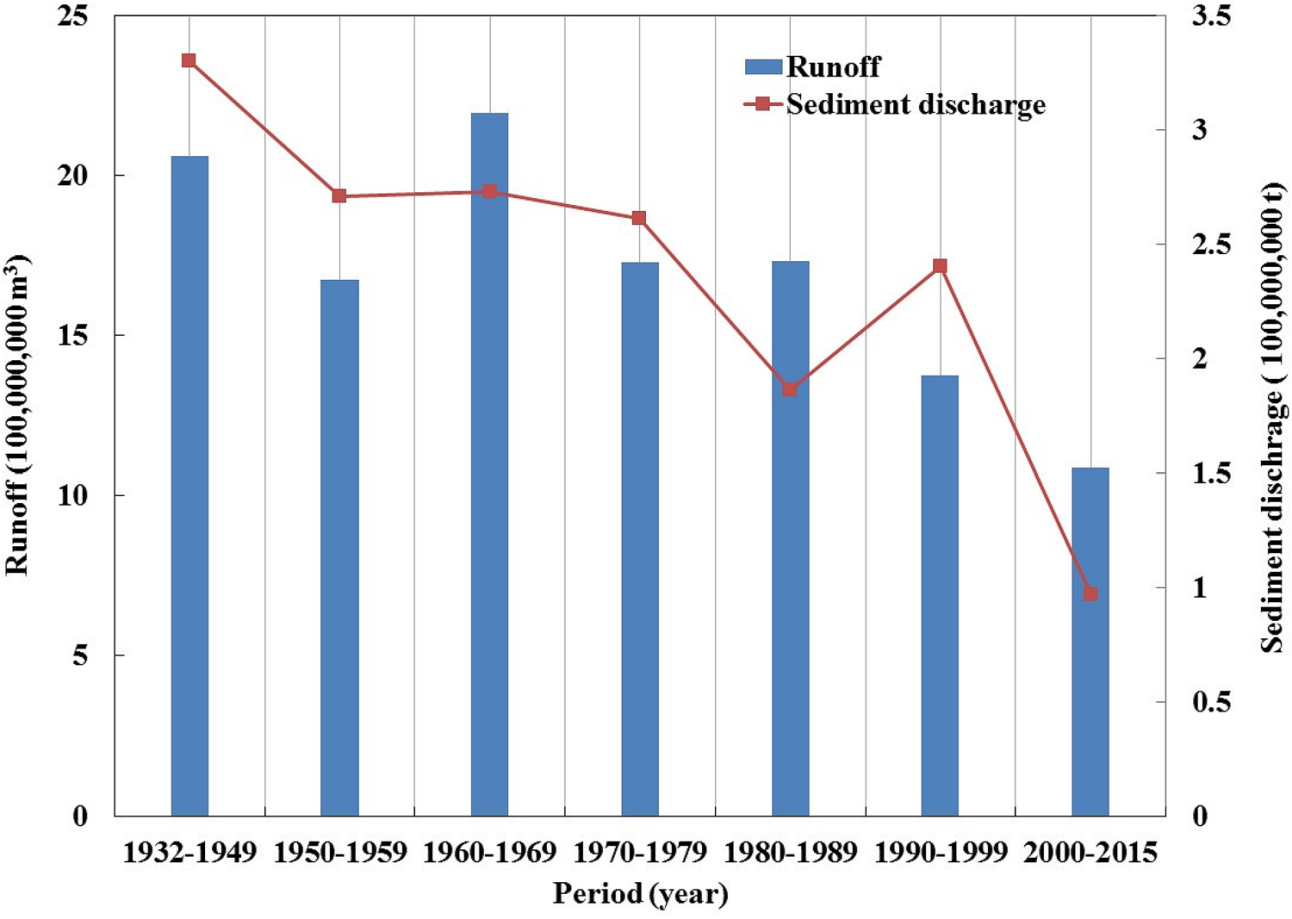
Variations in the average annual runoff and sediment discharge at the Zhangjiashan Station, Jinghe River Basin, during different periods.

**Figure 6:**
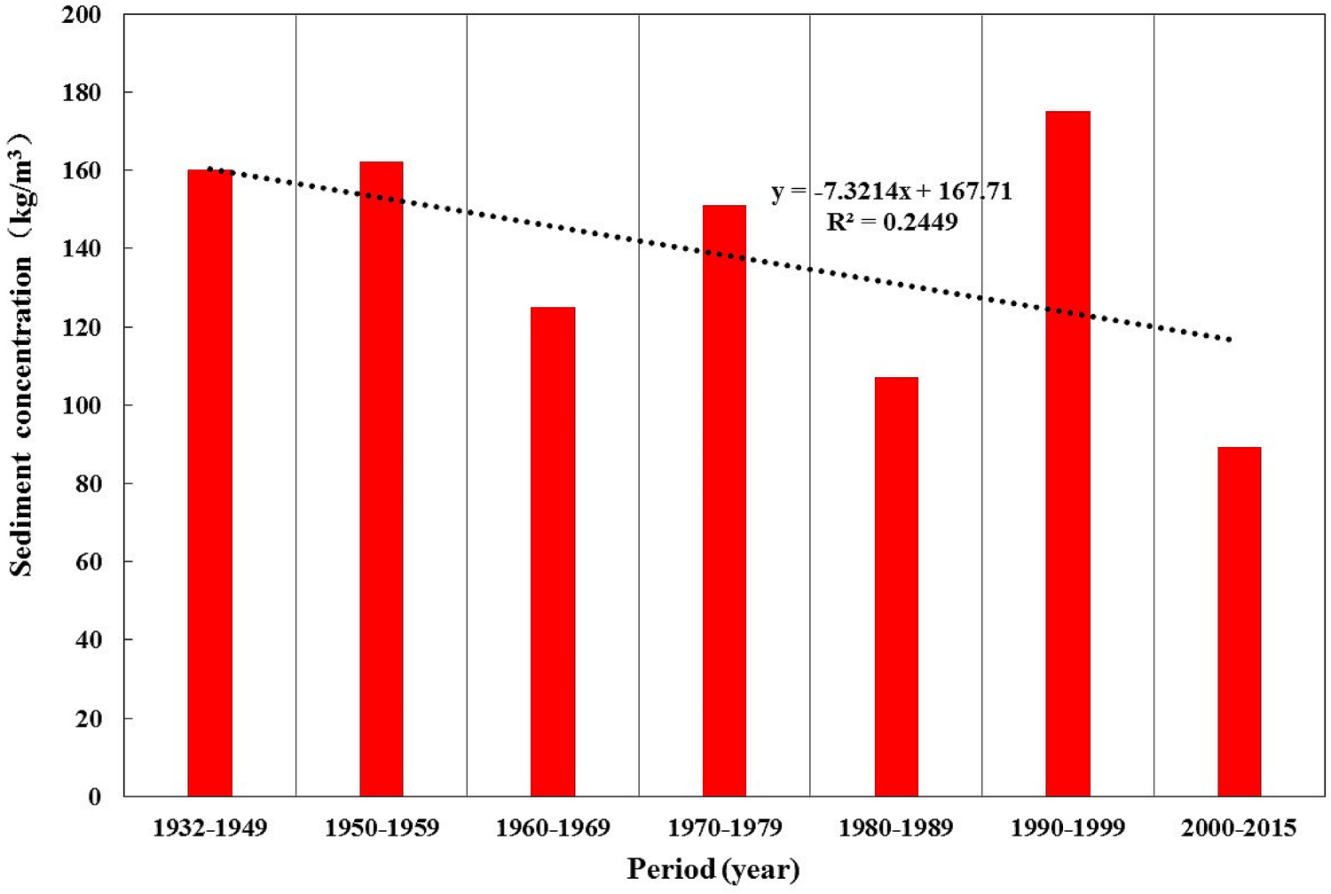
Variations in the average annual sediment concentration at the Zhangjiashan Station, Jinghe River Basin, during different periods.

Figure 5 depicts the variations in runoff and sediment discharge at Zhangjiashan Station during different periods. The average annual runoff was ~ 2,062,000,000 m^3^ before the 1950s, remained stable during the 1950s, 1970s, and 1980s, and peaked in the 1960s. The average annual runoff decreased significantly in the 1990s, and especially from 2000 to the present. The average annual sediment discharge was the highest before the 1950s, remained stable from the 1950s to 1970s, decreased in the 1980s, increased in the 1990s, and decreased again with a decreased runoff from 2000 to the present. The average annual sediment concentration in the 1960s and 1980s, as well as since 2000, was relatively low, whereas that in the other periods was comparable to the average annual sediment concentration. The sediment concentration fluctuated without any evident trends (Fig. 6).

**Figure 7:**
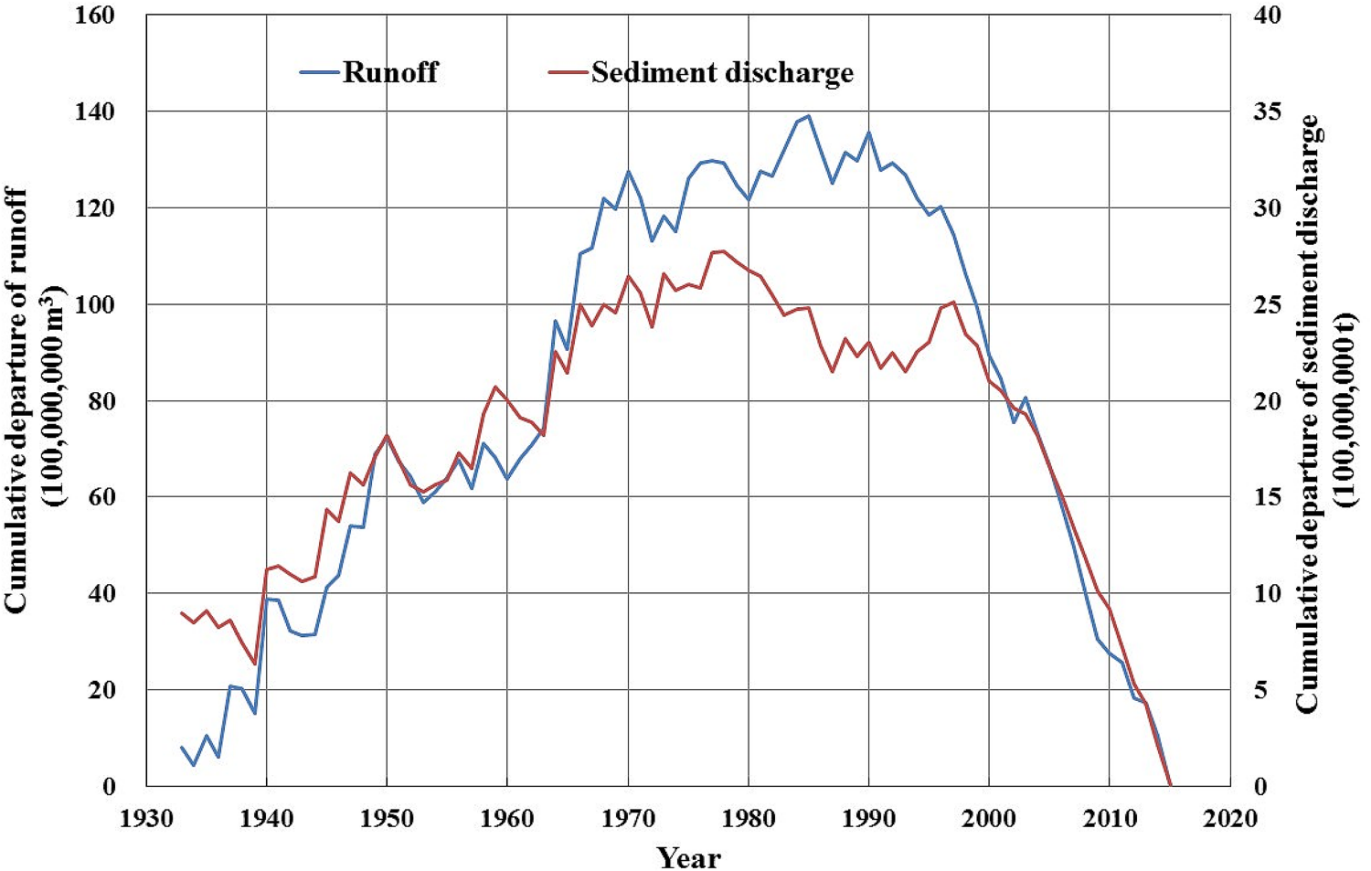
Cumulative departure curves of the runoff and sediment discharge at the Zhangjiashan Station, Jinghe River Basin, from 1930 to 2020.

**Figure 8:**
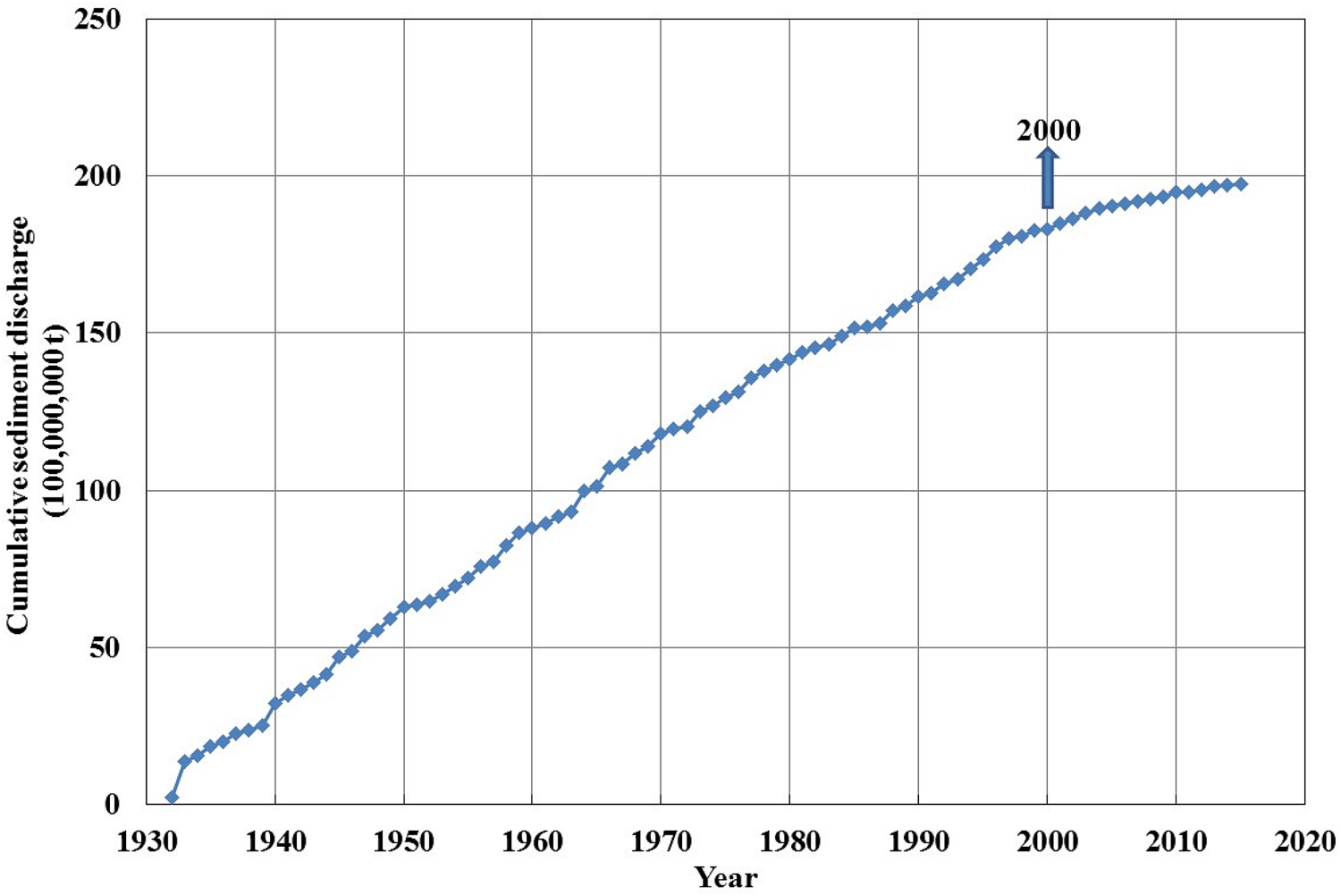
Cumulative curve of the sediment discharge at the Zhangjiashan Station, Jinghe River Basin, from 1930 to 2020.

Figure 7 depicts the cumulative departure curves of the runoff and sediment discharge at Zhangjiashan Station. Both curves trend downward from 2000 onwards, suggesting that the recent runoff and sediment discharge values in the Jinghe River Basin were low. Figure 8 depicts the cumulative curve of the sediment discharge. The cumulative sediment discharge since 2000 decreased, also suggesting that recent sediment discharge was low.

### Sediment reduction analysis

#### Sediment reduction by water diversion works

Water diversion works reduce sediment mainly by intercepting it via reservoirs and diverting it along with the water. As of 2015, the total capacity of completed reservoirs in the Jinghe River Basin was ~ 900,000,000 m^3^, including an effective regulating capacity of ~ 230,000,000 m^3^ and a sediment retention capacity of ~ 670,000,000 m^3^. Based on the reservoir sedimentation data, the average annual sediment retention by reservoirs in the basin from 1956–1999 was ~ 13,400,000 t.

As reservoir sedimentation continued, certain reservoirs lost much of their capacity and were thus unable to intercept sediment. The average annual sediment retention by reservoirs from 2000–2015 decreased annually by ~ 2,900,000 t, on average, to ~ 10,500,000 t (Table 2)^45^.

**Table 2:** Variations in the sediment reduction due to reservoirs in the Jinghe River Basin during different periods (Source: Yellow River Basin Monitoring Centre of Water-Soil Conservation and Eco-Environment^46^).

Average annual sediment retention by reservoir (100,000,000 t)		Average annual sediment reduction attributable to sediment retention by reservoir (100,000,000 t)
1956–1999 (long series)	2000–2015 (recent period)	
0.134	0.105	– 0.029

Based on the water and sediment diversion data from the basin, the average sediment concentration in the diverted water was approximately 50 kg/m^3^. Recent increases in water diversion have led to an average annual sediment discharge decrease of ~ 4,900,000 t (Table 3).

**Table 3:** Sediment reduction due to increased water diversion.

Water and sediment diversion	1956–1999 (long series)	2000–2015 (recent period)
Average annual water diversion (100,000,000 m^3^)	1.56	2.53
Average annual sediment diversion (100,000,000 t)	0.078	0.127
Increase in average annual water diversion, recent period (100,000,000 m^3^)		0.97
Decrease in average annual sediment due to increased water diversion (100,000,000 t)		0.049

These results suggest that, compared to the long time series, the average annual sediment retention by reservoirs in the basin since 2000 has decreased by ~ 3,000,000 t while the average annual sediment diversion has increased by ~ 5,000,000 t, leading to a net decrease of ~ 2,000,000 t in the average annual sediment discharge.

#### Sediment reduction by soil and water conservation works

**Figure 9:**
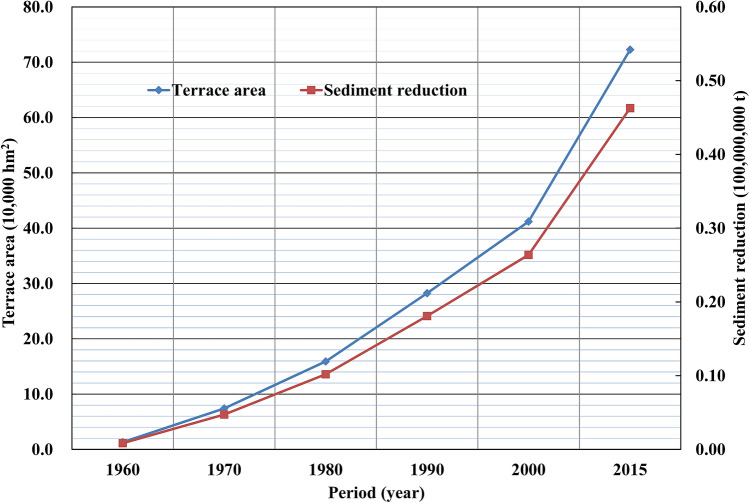
Relationship between the terrace area (blue diamonds) and sediment reduction (red squares) from 1960 to 2015.

**Figure 10:**
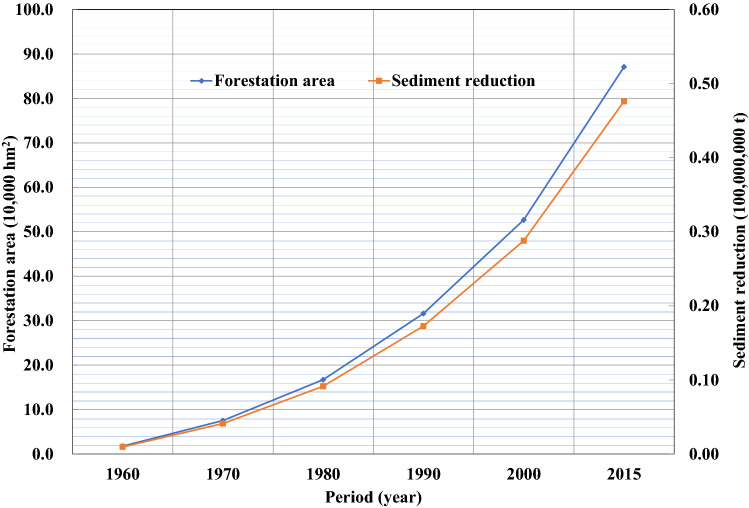
Relationship between forestation area (blue diamonds) and sediment reduction (red squares) from 1960 to 2015.

**Figure 11:**
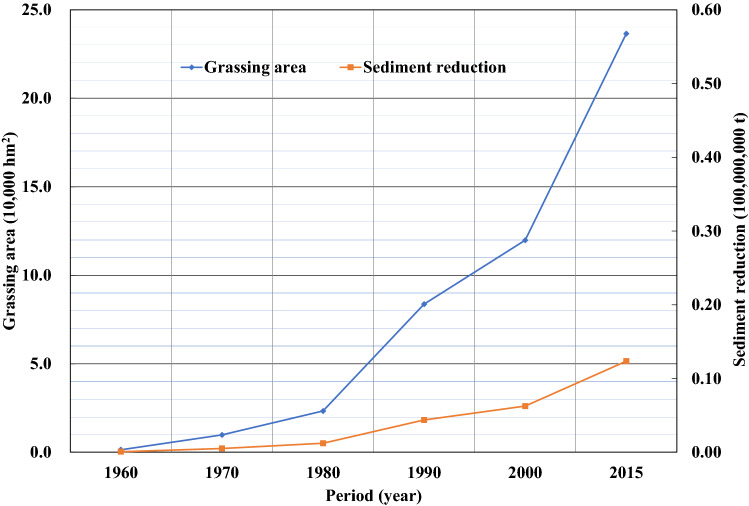
Relationship between grassing area (blue diamonds) and sediment reduction (red squares) from 1960 to 2015.

**Figure 12:**
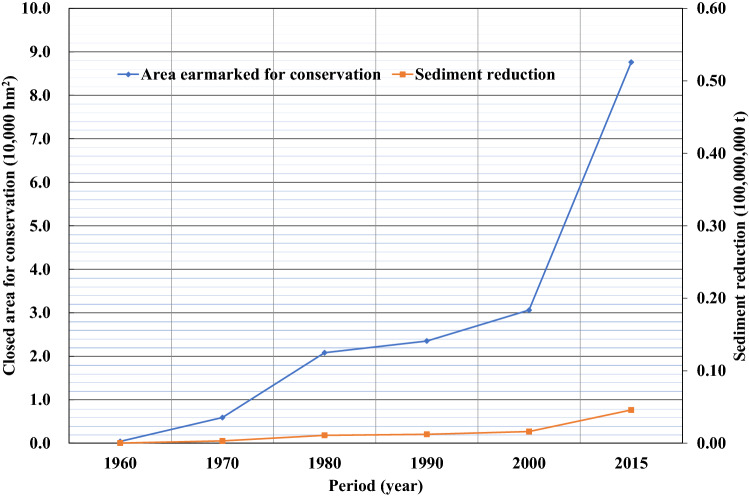
Relationship between areas closed for conservation (blue diamonds) and sediment reduction (red squares) from 1960 to 2015.

**Figure 13:**
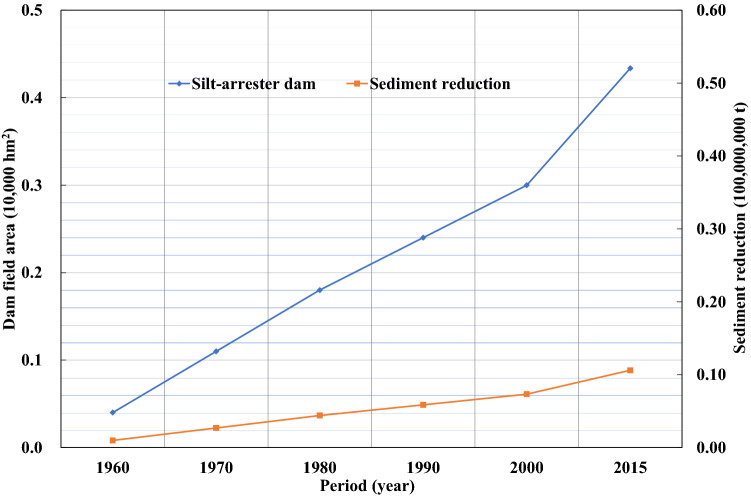
Relationship between dam field area (blue diamonds) and sediment reduction (red squares) from 1960 to 2015.

Since the 1970s, Gansu and Shaanxi provinces have actively engaged in soil and water conservation by returning farmland to forests or grassland and by creating enclosures for large-scale afforestation in the Jinghe River Basin, which has led to a continued increase in dam fields, terraces, forests, and grasslands. Figures 9–13 illustrate the relationship between the conservation areas and sediment reduction. Sediment reduction increased with the growth of the conservation areas. The total soil and water conservation area (sum of the conservation areas) in the Jinghe River Basin was ~ 166,000 hm^2^ in 1970 and increased 10.6-fold to ~ 1,922,000 hm^2^ in 2015. The most notable increase was ~ 830,000 hm^2^ from 2000–2015, which represents an average annual growth of ~ 55,000 hm^2^. Among the conservation measures, terraces and forested areas account for 37.7–44.6% and 44.6–48.2% of the total conservation area, respectively. Based on calculations conducted in this study, the soil and water conservation measures implemented between 1956 and 1999 contributed to an average annual sediment reduction of ~ 19,000,000 t while measures from 2000–2015 led to a reduction of ~ 96,000,000 t, amounting to an increase of ~ 77,000,000 t attributable to the recent implementation of soil and water conservation works.

#### Correlation between variations in annual rainfall and sediment discharge

Rainfall is distributed unevenly over time and space, varies widely from one year to another, and is highly concentrated in a given year. The average annual rainfall for the period from 1956–2015 in the Jinghe River Basin was 506.1 mm while that for 1956–1969 was 534.6 mm, 5.6% higher than the multi-year average. The average annual rainfall from 1970–1979 and 1980–1989 (490.2 and 491.4 mm, respectively) was comparable and approximately 3% lower than the multi-year average. The value from 1990–1999 was the lowest at 474.2 mm, 6% lower than the multi-year average, while that from 2000–2015 was 522.2 mm, 3% higher than the multi-year average. Compared with the annual rainfall from 1980–1989, the average annual rainfall from 1990–1999 decreased by 3.5%, with an increase in the soil and water conservation area, but the sediment discharge increased by 28%. Therefore, the effect on sediment yield cannot be attributed to the average annual rainfall. Table 4 lists the average annual rainfall and sediment discharge for different periods in the Jinghe River Basin.

**Table 4:** Average annual rainfall and sediment discharge during different periods in the Jinghe River Basin.

	Period						
	1956–1969	1970–1979	1980–1989	1990–1999	2000–2013	1956–1999	1956–2015
Average annual rainfall (mm)	534.6	490.2	491.4	474.2	522.2	501.0	506.1
Average annual sediment discharge (100,000,000 t)	2.99	2.60	1.86	2.38	1.05	2.50	2.15

#### Short-duration rainfall-induced sediment discharge variations

The average annual sediment discharge from 1956–1999 in the Jinghe River Basin was ~ 250,000,000 t, while that from 2000–2015 decreased by ~ 153,000,000 t to ~ 97,000,000 t. Water conservation works led to an average annual sediment reduction of 2,000,000 t. Soil and water conservation works led to a further decrease of ~ 77,000,000 t in the average annual sediment. After subtracting the sediment reduction increment attributable to water conservation projects and soil and water conservation works, the remaining average annual sediment reduction attributable to the variations in rainfall amounted to ~ 74,000,000 t. Most sediment in the Jinghe River Basin originates from the loess plateau and hill gully areas because of heavy rainfall. To analyse the effect that rainfall has on the sediment yield, the variations in hourly rainfall were examined.

**Figure 14:**
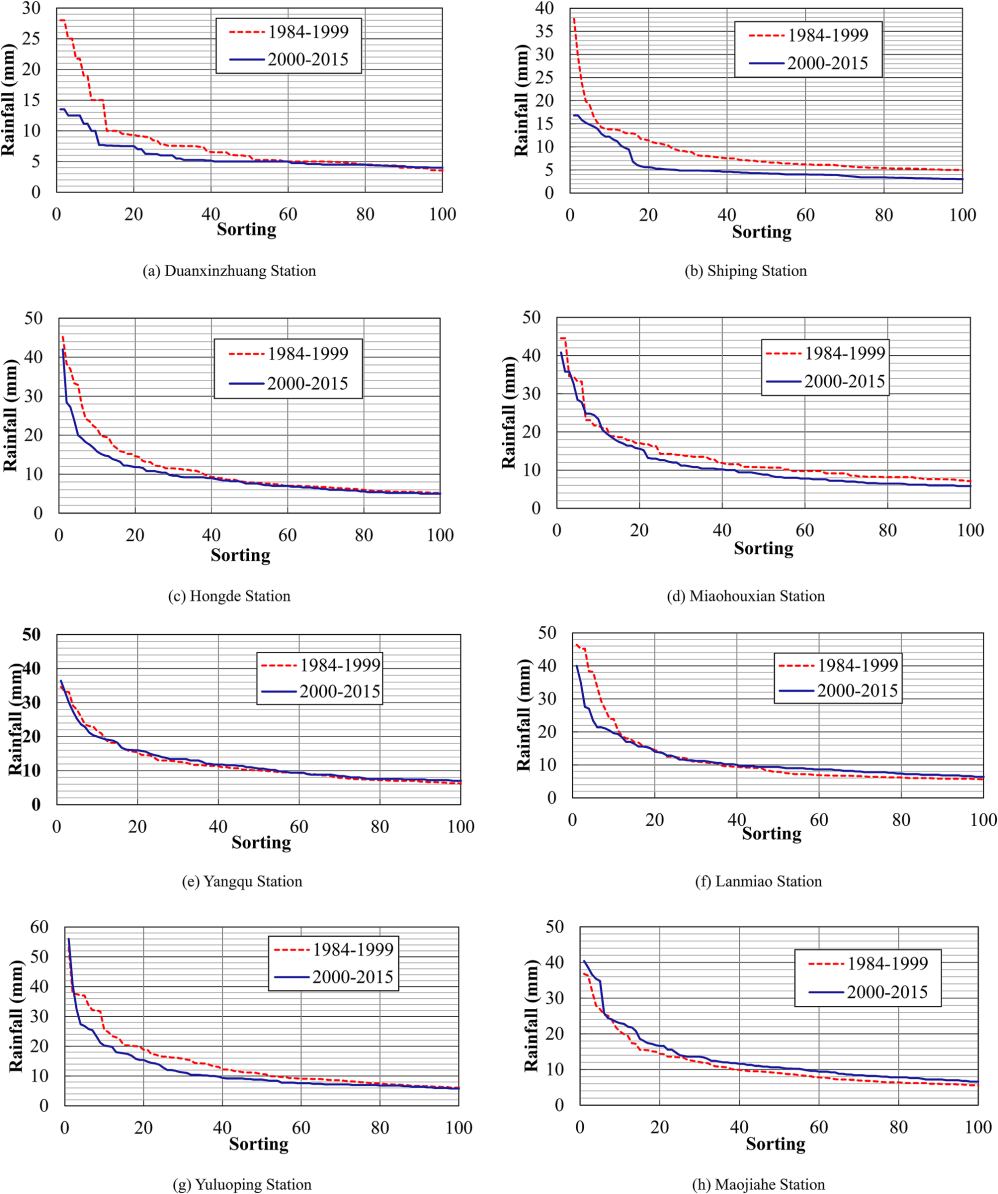
Sorting of the 1-h rainfall data collected from typical rainfall stations in the Jinghe River Basin.

One-hour rainfall data were collected from four stations (Duanxinzhuang, Hongde, Yangqu, and Yuluoping) in the Malian River Basin and four stations (Shiping, Miaohouxian, Lanmiao, and Maojiahe) in the Puhe River Basin, where the sediment discharge was high (Fig. 3). To compare the 1-h rainfall data from 2000–2015 and 1984–1999, data from each station were sorted in descending order and plotted (Fig. 14). The curves show that, compared with the rainfall data from 1984–1999, there was a significant decrease in the 1-h rainfall from 2000–2015 at seven of the eight stations in the primary sediment yield areas. The only exception is the Maojiahe Station on the lower Puhe River. Therefore, the rainfall patterns in the primary sediment yield areas of the Jinghe River Basin have changed since 2000. Despite an increasing annual rainfall, heavy rainfall, which greatly affects the sediment yield, has experienced a significant overall decrease.

### Sediment yield and uncertainty analysis

#### Calculation results for the sediment yield in the Jinghe River Basin

**Figure 15:**
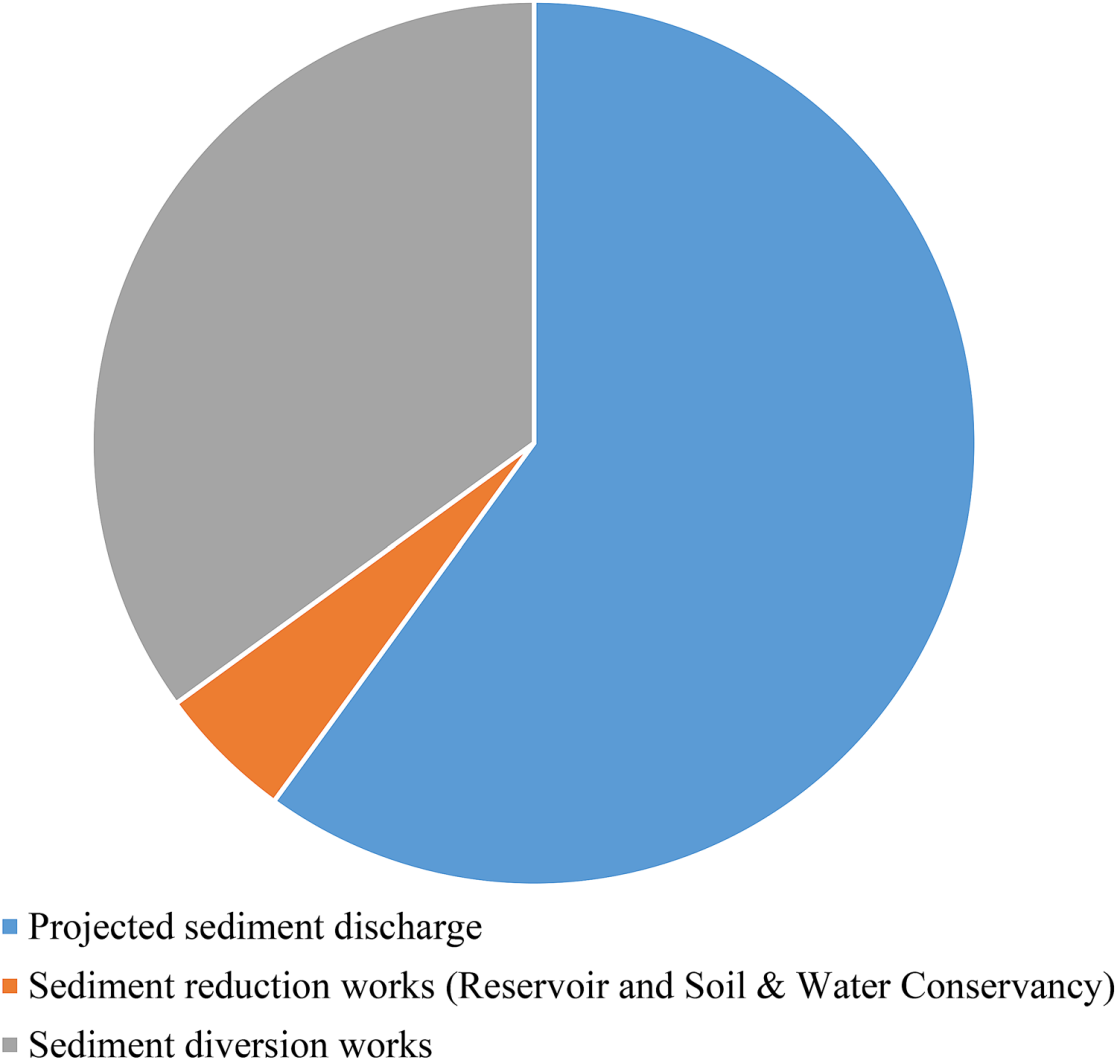
Calculation results for the sediment yield in the Jinghe River Basin.

Figure 15 depicts the calculation results for the sediment yield in the Jinghe River Basin. Based on Fig. 5, the natural average annual sediment discharge in the basin is ~ 270,000,000 t and the future average annual sediment discharge is projected to be ~ 161,000,000 t. Among them, the average annual sediment reduction in the conservation area was ~ 95,900,000 t and the average annual sediment diversion was ~ 12,700,000 t.

Existing silt-arrester dams are losing their sediment retention capability, where certain dams have reached their full sedimentation capacity, limiting their future sediment retention capability. If silt-arrester dams lose their ability to retain sediment after the reservoir reaches its sedimentation capacity and heavy rainfall occurs in the basin, high sediment discharge will be inevitable along the Jinghe River. Additionally, vegetation cover in the basin is 55%, and future sediment reduction by soil and water conservation works is also likely to decline. All these factors add a variety of uncertainties to the calculation results.

#### Analysis of uncertainties in the calculation results

Since the 1950s, sediment reduction works and economic and social water consumption in the Jinghe River Basin have gradually increased. Both sediment reduction works (through sediment retention and water storage) and water and sediment diversion projects have an important impact on runoff and sediment reduction in the Jinghe River. The former directly intercepts sediment moving into the Jinghe River. The latter draws water from the Jinghe River for economic and social use, which simultaneously reduces the runoff and sediment of the Jinghe River. Despite their small sediment retention capacity, these projects are typically small- or medium-scale and numerous, and many are built by local governments and farmers themselves. Determining the time of completion and time nodes of operation for these projects is therefore difficult, which may cause deviations in the calculation results.

Additionally, the Jinghe River Basin is a region in the middle reaches of the Yellow River characterised by frequent storm events. Short-duration heavy rainfall often leads to major floods that scour slope surfaces and gullies during runoff, removing large amounts of sediment. Under the effects of global climate change, short-duration heavy rainfall in the basin is likely to become more intense and frequent, increasing sediment discharge. Given the numerous contributors to sediment variation in the basin, especially frequent extreme weather events, there should not be a heavy reliance on soil and water conservation works.

Owing to the above two factors, the analysis of sediment reduction in the Jinghe River Basin is complicated. To avoid large deviations in the calculation results, from the 1990s to the present, four Chinese agencies or project teams have conducted continuous studies on the causes of post-1960 sediment reduction in the Jinghe River Basin and the forecast of future sediment yield.

Table 5 presents the outcomes of these previous studies on the forecasted trends in sediment discharge variations. The forecasted sediment discharge in different studies varied from ~ 85,000,000 to ~ 217,000,000 t^46–49^.

**Table 5:** Predicted annual sediment discharge in the Jinghe River (Sources: Yao et al.^47^; Liu et al.^48^; Yellow River Conservancy Commission^49,50^).

Studies	Period	Sustainable sediment reduction in Jinghe River Basin (100,000,000 t)	Design sediment into reservoir (100,000,000 t)
National 11th Five-Year Plan for Science and Technology Grant	1997–2006	0.535	2.166
National 12th Five-Year Plan for Science and Technology Grant	2007–2014	1.632–1.849	0.851–1.068
White Paper: Study on Variation in Yellow River Runoff and Sediment	2000–2012	0.852	1.848
Comprehensive Plan for Yellow River Basin (2012–2030)	2030	0.6	2.1
Present study	2000–2015	1.086	1.614

Based on Table 4, the future average annual sediment discharge in the Jinghe River will range from ~ 160,000,000 to ~ 210,000,000 t, and will continue to play an important role in the Weihe and Yellow River sedimentation levels, possibly leading to dangerous flood releases in the river channel. The results of this study highlight the necessity of implementing large sediment trapping works in the Jinghe River Basin.

## Discussion

Human activity and climate change are the two major factors that affect the hydrological processes of river basins, which manifest themselves in runoff and sediment discharge. Specifically, both human activity and climate change affect runoff and sediment discharge in river basins. To understand the reason for decreases in the runoff and sediments in the Jinghe River Basin, an attribution analysis of runoff and sediment variations was conducted, focusing on the contributions of human activities and climate change. It quantitatively evaluated the impact on water and sediment changes in the Jinghe River due to climate change, vegetation restoration, water and soil conservation works, and landscape patterns.

The Jinghe River Basin has a natural average annual sediment discharge of 270 million t, forming the main source area of sediment in the Yellow River. Since the 1950s, large-scale efforts in water and soil conservation and management, such as transforming terraces, planting trees and grasses, building dams, returning farmland to forests, closing mountains and banning grazing, have been implemented in the Jinghe River Basin and have significantly changed the land use and vegetation cover in the basin. Numerous existing studies suggest that, throughout the past 60 years, changes in the underlying surface conditions caused by human activity are a key factor in the runoff and sediment reduction across the Loess Plateau. However, the authors determined that changes in the water and sediment regimes in the river basin are a combined result of changes in the climate and underlying surfaces. The impact that human activity has on the runoff and sediment yield is mainly embodied in the direct effects of structural measures and the production and consumption of domestic water, as well as indirect effects associated with changes in the underlying surface. Climate change affects the hydrological cycle of a river basin. Climate change, characterised by changes in precipitation and temperature, plays an important role in runoff formation, geographical distribution, and sediment carrying capacity.

Numerous factors influence the runoff and sediment variations in rivers. The mechanistic relationships among the climate, underlying surfaces, runoff, and sediment discharge are complicated. Any method may cause errors due to improper considerations^50^^.^ To reduce this error, the authors proposed the adoption of a variety of methods to compare and verify the calculation results. For example, statistical analysis was conducted. The statistical relationship between runoff and sediment discharge and climatic variables, such as precipitation, was established for the baseline period with negligible impacts from human activity. The runoff and sediment discharge during the impact period was predicted and compared with the measured value to qualitatively analyse the causes of such variations. Based on this, the hydrological model was used as a tool to simulate natural runoff and sediment discharge. By comparing the measured and simulated natural runoff and sediment discharge during the impact period, the contributions of various factors to runoff and sediment processes in the river basin during the impact period were separated to achieve a quantitative attribution analysis of the runoff and sediment variations.

Expanding our understanding of basin runoff and sediment discharge mechanisms in a changing environment is necessary. Therefore, future studies must be conducted with respect to three aspects:

(1) Basin runoff and sediment characteristics and the temporal and spatial evolution of extreme events. Quantitative research should be conducted on the temporal and spatial characteristics of the runoff-sediment evolution in river basins, especially the nonlinear characteristics of the runoff-sediment relationship, to examine the characteristics and mechanisms of runoff-sediment variations.

(2) A multi-factor comprehensive analysis of the impact mechanism of runoff and sediment variations. Future studies should investigate the quantitative contributions and temporal and spatial patterns of factors, such as vegetation restoration, structural measures, terrain, and soil, as well as climate change and human activity (i.e. socio-economic development and ecological construction policies), to variations in the runoff and sediment. A precipitation-runoff-sediment model that couples the dynamic characteristics of land cover and climate change can be established to reveal the mutual feedback mechanism of ecological restoration and runoff-sediment evolution.

(3) Scenario simulation and trend prediction of future runoff and sediment dynamics. As the sediment retention capacity of structural measures, such as silt-arrester dams, gradually declines, the trends of runoff and sediment dynamics under the scenarios of climate change, socio-economic development, and ecological construction projects must be predicted to provide strategic recommendations that will allow a sustainable hydrological system in the Jinghe River Basin to be implemented. Such research will play an important role in controlling a rebound of sediment discharge to the Yellow River.

## Conclusions

Since the 1950s, the Yellow River Conservancy Commission has promoted various soil and water conservation works in the Jinghe River Basin, including modifying terraced fields, planting trees and grass, building dams, returning farmlands to forests, and creating enclosures. These conservation practices have produced satisfactory initial results, reducing sediment discharge in the Jinghe River to a certain degree. Since 2000, the average annual sediment discharge in the river has decreased by ~ 153,000,000 t (61.2%) to ~ 97,000,000 t from that during the period from 1956–2000, which suggests that current conservation practices in the basin have been successful, such that studies have consequently forecasted a sustainable reduction in sediment discharge into the river. From this perspective, there is no urgent need to construct new reservoirs for flood control and sediment reduction. This study investigated the variations in sediment retention by reservoirs, water diversion works, soil and water conservation measures, and rainfall variations and their intensities during different periods to comprehensively analyse the causes of the recent significant reductions in sediment discharge in the Jinghe River Basin. The results of this study suggest that water diversion works, soil and water conservation measures, and variations in rainfall are the major contributors to the changes in sediment discharge. Areas of primary sediment yield in the basin have shown notable decreases in heavy rainfall since 2000, which has significantly contributed to the reduced sediment discharge in the Jinghe River. Analysis of a large amount of measured data using the soil and water conservation and rainfall analysis methods showed that soil and water conservation works caused a decrease in sediment of ~ 74,000,000 t, representing 48.4% of the total reduction. Sediment retention by reservoirs and water and sediment diversion works resulted in a sediment decrease of ~ 2,000,000 t, representing 1.3% of the total reduction. Climate change may lead to frequent extreme weather events in the future. Future heavy rainfall is likely to increase in the Jinghe River Basin, which will result in a higher sediment discharge. Therefore, construction of the Dongzhuang Reservoir should remain an essential consideration in the Yellow River runoff and sediment control system. The natural average annual sediment discharge in the basin was ~ 270,000,000 t.

Given the topographic and geomorphic features of the basin, gully erosion has remained unchanged and is likely to continue to do so in the future. Current vegetation cover in the basin is 55%, which is likely to complicate the implementation of soil and water conservation works. Furthermore, silt-arrester dams are likely to play a reduced role in sediment reduction once they reach full capacity. Sustainable average annual sediment reduction by soil and water conservation works is predicted to be ~ 60,000,000– ~ 109,000,000 t, while the average annual sediment discharge in the river is projected to be ~ 160,000,000– ~ 210,000,000 t. The results of this study are of great significance to regulatory agencies and the public because they provide important technical support for the development and management of the Jinghe, Weihe, and Yellow rivers, the regulation and operation of existing water conservation projects, and decision-making tools for proposed major hydro projects. They also offer insight into the current variations in the runoff and sediment values.

## Methods and data

### Sediment reduction analysis method

This section presents the methods used to calculate sediment reduction caused by the major contributors, i.e., reservoir works, water diversion works, soil and water conservation works, and rainfall.

#### Sediment reduction by reservoir works

Reservoir works reduce sediment by impounding and retaining the sediment. Recent variations in sediment reduction due to reservoir works were analysed according to the variations in the average annual sediment deposition in the reservoirs of the basin during different periods.

The average annual sediment reduction by various reservoirs can be calculated by dividing the accumulated sediment in each reservoir during a certain period by the number of years:

1 $$ WS_{r} = \sum\limits_{1}^{n} {D_{i} /N} , $$

where $D_{i}$ is the accumulated sediment (100,000,000 t) in a reservoir during a certain period, *N* is the number of years in the period, and *WS*_*r*_ is the average annual sediment reduction (100,000,000 t) in the period by all reservoirs in the basin.

The Hydrological Bureau under the Yellow River Water Conservancy Commission annually measures and calculates the deposition of sediment in all reservoirs in the Yellow River Basin. Two methods can be used, namely, the topographic method and the section method. In the topographic method, the area enclosed by contour lines on the topographic map of the reservoir area is measured to calculate the reservoir volume. The cumulative deposition of sediment during a specific period is the difference between the current and the previous reservoir volume. The topographic method requires closed contour lines on the map. In reality, however, the contour lines cannot be closed due to the presence of farmland, houses, and other artificial structures in the reservoir area, resulting in measurement errors. Therefore, the section method is mainly used at present. Here, *M* test sections were deployed in the reservoir area, and the test section data were used to calculate the total storage capacity of the reservoir in sections by period, as follows:

2 $$ V_{i} = \sum\limits_{m = 1}^{M - 1} {V_{i,m} } . $$

The difference in the storage capacity measured twice is the cumulative deposition of sediment in reservoir $D_{i}$:

3 $$ D_{i} = V_{i - 1} - V_{i} , $$

where *V*_*i*_ is the storage capacity measured at the end of period *i* and *V*_*i,m*_ is the storage capacity measured in section *m* – 1.

#### Sediment reduction by water diversion works

During water diversion, a certain amount of sediment is diverted, along with water, and is deposited in irrigation areas, resulting in a decrease in the volume of the sediment in the river channel. The average annual sediment reduction by water diversion works can be calculated by multiplying the average annual water diversion in different periods in the Jinghe River Basin by the average annual sediment concentration in the water diversion period, as follows:

4 $$ WS_{d} = \sum\limits_{1}^{n} {W_{di} /N \times \overline{S}} /{1}000, $$

where $W_{di}$ is the cumulative water diversion (100,000,000 m^3^) in the basin in the water diversion period, *N* is the number of years in the period, $\overline{S}$ is the average annual sediment concentration in the period (kg/m^3^), and $WS_{d}$ is the average annual sediment reduction (100,000,000 t) in the basin during the period. Recent variations in sediment discharge caused by water diversion works were analysed according to the variations in the average annual water diversion in the basin in different periods.

#### Sediment reduction by soil and water conservation works

A commonly used method to compute the sediment reduction by soil and water conservation works is to multiply the area subject to the soil and water conservation works, such as terracing, forestation, grassing, creating enclosures, and constructing silt-arrester dams, by the sediment reduction by each measure per unit area, followed by their summation, as follows:

5 $$ WS_{SC} = \sum\limits_{1}^{n} {F_{i} \times S_{j} /10^{8} ,} $$

where *S*_*j*_ is the sediment reduction due to each soil and water conservation measure (t/hm^2^), published by the soil and water conservation monitoring institutions in each basin based on the analysis of the long-term observation data, *F*_*i*_ is the area subjected to each measure (hm^2^), and *WS*_*SC*_ is the comprehensive sediment concentration for each measure (100,000,000 t). The variations in sediment reduction by soil and water conservation works were analysed based on the variations in the soil and water conservation areas in the basin during different periods.

#### Analysis of rainfall-induced sediment yield

The deduction method was adopted to analyse the rainfall-induced variations in the sediment yield. Recent variations in sediment reduction attributable to reservoirs, water diversion, and soil and water conservation works were computed and deducted from the measured sediment reduction in recent years (2000–2015):

6 $$ \Delta WS_{p} = \Delta WS_{t} - \Delta WS_{r} - \Delta WS_{d} - \Delta WS_{sc} , $$

where $\Delta WS_{t}$ is the recently measured sediment reduction (100,000,000 t), $\Delta WS_{r}$ is the recent variation in the sediment reduction (100,000,000 t) caused by variations in the sediment retention due to reservoir works, $\Delta WS_{d}$ is the recent variation in sediment reduction (100,000,000 t) caused by variations in water diversion, $\Delta {\text{WS}}_{{{\text{SC}}}}$ is the recent variation in sediment reduction (100,000,000 t) caused by variations in the soil and water conservation area, and $\Delta WS_{p}$ is the recent variation in the rainfall-induced sediment yield caused by variations in rainfall.

### Sediment yield calculation method

Figure 6 depicts the computational process for the sediment calculation. First, a reduction calculation of the natural runoff was performed as follows:

7 $$ W_{0} = W_{m} + W_{cum} + W_{s} + W_{e} + W_{SC} , $$

where *W*_0_ is the natural runoff, *W*_*m*_ is the measured runoff, *W*_*cuw*_ is the industrial water consumption in the basin, *W*_*s*_ is the water retention by reservoirs, *W*_*e*_ is the water evaporation and seepage losses, *W*_*sc*_ is the water reduction by soil and water conservation, and *W*_0_ is the natural water volume in the basin. All these terms are in 100,000,000 m^3^.

Second, the runoff-sediment relationship in the natural state was established based on the measured runoff and sediment data in periods with negligible human activity, as well as when the underlying surface was in a nearly natural state. Natural sediment discharge was calculated using the relationship between runoff and sediment discharge. According to the observation data from the basin for the past 35 years, runoff was closely related to sediment discharge. Given China’s climatic conditions and economic growth, the basin was nearly in a natural state up to 1960 because human activity had a minor impact on runoff and sediment discharge. Based on the runoff and sediment discharge measurements at Zhangjiashan Station from 1932 to 1960, the relationship between the natural runoff and sediment discharge was established as *WS*_0_ = *f*(*W*_0_). Natural sediment discharge in the basin was calculated considering the restored natural runoff.

Third, the natural sediment discharge was calculated using the natural runoff results and the runoff-sediment relationship. Based on the major contributors to sediment reduction in the basin, the future sustainable sediment reduction was calculated as the sum of sediment reduction due to reservoirs, water diversion, and soil and water conservation measures. Sediment reduction caused by variations in rainfall was limited to certain periods. For example, recent reduced heavy rainfall has led to a decreased rainfall-induced sediment yield and consequently a decreased sediment discharge. However, according to forecasts by the *Intergovernmental Panel on Climate Change* (2014)^50^, extreme weather and heavy rainfall events are likely to increase in the future. The reduction in sediment due to variations in rainfall was calculated as follows:

8 $$ WS_{d} = WS_{r} + WS_{d} + W_{SC} , $$

where *WS*_*r*_ is the future sediment reduction caused by reservoir works, i.e., the sum of the sediment retention potential of the remaining capacity of the existing reservoirs and that of planned future reservoirs; *WS*_*d*_ is the sediment reduction caused by future water diversion works, which can be obtained by multiplying the water diversion in the basin forecasted according to the social and economic development by the average sediment concentration in the water diversion period; *WS*_*sc*_ is the future sediment reduction caused by soil and water conservation, obtained from areas subject to existing and planned soil and water conservation works and the corresponding sediment reduction rates; and *WS*_*d*_ is the forecasted value of sediment reduction in the basin. All these terms are in 100,000,000 t.

Fourth, the sustainable sediment reduction in the basin was calculated considering variations in the contributions to sediment reduction in a future period and their effect. Future sediment discharge in the basin is the difference between the natural and future sediment reduction, as follows:

9 $$ WS_{f} = WS_{0} - WS_{d} , $$

where *WS*_0_ is the natural sediment discharge in the basin, *WS*_*d*_ is the forecasted sediment reduction in the basin, and *WS*_*f*_ is the forecasted sediment discharge in the basin. All these terms are in 100,000,000 t.

Finally, future river sediment discharge was obtained by subtracting the future sustainable sediment reduction from the natural sediment discharge.

### Data acquisition

#### Hydrological data

A total of 28 hydrometric stations and 190 rainfall stations are located along the main stream and tributaries of the Jinghe River to effectively monitor rainfall, runoff, and sediment in the basin.

Zhangjiashan Station, located at the outlet of the Jinghe River Basin, has a catchment area of 432,160,000 km^2^, covering 95% of the total area of the basin. Few hydrometric and rainfall stations were operational in this basin before 1956, and hence incomplete data were collected. Analyses in this study were based on data from the Zhangjiashan Station from 1956–2015. At this station, the cross-sections in the main stream and Jinghui Canal (a water diversion canal) were hydrologically measured to determine the discharge, sediment transport rate, and sediment concentration.

#### Engineering data

Data on sediment reduction due to reservoir works and terraces, forests, grasslands, enclosures, and dams in the basin were based on the results of the National Water Resources Census and official data collated by the Upper and Middle Yellow River Bureau of the *Yellow River Conservancy Commission*. These data are thus accurate and reliable.

For data collection and erosion–deposition calculations, DL/T 5089–1999 "Specification for Sediment Design of Hydropower and Water Conservancy Projects" provided that "The calculated results of erosion and deposition should be compared with the measured data for several years of operation. If the amount and location of sedimentation are 70% consistent, and the elevation of sedimentation in the reservoir differs by 1 to 2 m, then the calculated results are deemed reliable. For erosion–deposition calculation results, only reliability is considered".

Relevant data from the stations were systematically verified and collated by the Hydrological Bureau of the Yellow River Conservancy Commission and are therefore accurate and reliable.

## Data Availability

The data that supports the findings of this study are available upon request from the corresponding author. The data are not publicly available due to privacy or ethical restrictions.

## References

[1] Hu W (2016). The influence of human activities on the runoff and sediment load changes of Hanjiang River. Res. Soil Water Conserv..

[2] Liu X, He D (2012). A new assessment method for comprehensive impact of hydropower development on runoff and sediment changes. J. Geogr. Sci..

[3] Han B, Li N, Zeng C, Wang L (2015). Analysis of influence of large-scale water conservancy project on variation characteristics of water and sediment in middle and lower reaches of Yangtze River. J. Water Res. Water Eng..

[4] Wang Y, Shi H, Liu Q (2014). Influence of sediment trapping in reservoirs on runoff and sediment discharge variations in Yangtze River. Adv. Water Res..

[5] Gan C, Shi S, Zhu Y, Zhang L (2018). Impacts of hydropower engineering projects on the sediment yields in Hongshui River. Pearl River.

[6] Li, Y. X., Dang, S. Z., Dong, G. T. & Cheng, C. X. Response of runoff and sediment discharge to precipitation variation in the Kuye River Basin. In *Proceeding International Conference on Environmental Climate Change Sustainable Development.* May 28–29, Beijing, China, 358–364, 10.12783/dteees/eccsd2016/5854 (2016).

[7] Zhou P, Wen AB, Yan DC, Shi ZL, Guo J, Ju ZS, Zhang YL (2014). Changes in land use and agricultural production structure before and after the implementation of grain for green program in Western China—taking two typical counties as examples. J. Mt. Sci..

[8] Zhang, P. & Sun, W. Effect of comprehensive harnessing on water and soil conservation in Sanchuanhe river basin. *Proc. 2011 Int. Symp. Water Resources Environmental Protection.* May 20–22, Xi’an, China, 2475–2477, 10.1109/ISWREP.2011.5893377 (2011).

[9] Yang YY, Li ZB, Ren ZP, Gao HD (2017). The influence of human activities on the changes of water and sediment in different landforms. J. Sediment. Res..

[10] Jia Y (2017). Analysis of variation trends and cause of runoff and sediment in Yihe River Basin. Res. Soil Water Conserv..

[11] Yu, F. Study on Climate Change in Last 50 Years in Northeast China Region and Its Impact on Runoff and Sediment. Unpublished master’s thesis/doctoral dissertation. Northwest Agriculture & Forestry University, Xianyang, China (2011).

[12] Zhang Y, Hu C, Wang Y (2014). Analysis on variation characteristics and influential factors of runoff and sediment of Liaohe River Basin. Yangtze River.

[13] Liu, H. Study on Rainfall Variation and Human Activity Effect on Runoff and Sediment Characteristics in Upper Beiluo River. Unpublished master’s thesis/doctoral dissertation. Northwest Agriculture & Forestry University, Xianyang, China (2012).

[14] Ye C, Zhang ZD, Zhang J, Zhu R-X, Dong CW (2017). Analysis of runoff and sediment change and impact factor in the southern humid area river basin—a case study of Wuhua. Resour. Environ. Yangtze Basin.

[15] Zhang X, Liu M (2018). Effects of climate change and human activities on water and sediment discharge in Xiangjiang Basin. Res. Soil Water Conserv..

[16] Wang G, Li Z, Tian Y, Qu J, Xu J, Liu Z, Yan D (2017). Effects of rainfall intensity and land use on flow volume and sediment yield in Southwest Coteau of Henan Province. Eng. J. Wuhan Univ..

[17] Yan Q, Yuan C, Lei T, Lei Q, Zhang M, Su G (2014). Effect of rainstorm patterns and soil erosion control practices on soil and water loss in small watershed on loess plateau. Trans. Chin. Soc. Agr. Mach..

[18] Du H-Q, Xue X, Wang T (2015). Mapping the risk of water erosion in the watershed of the Ningxia-Inner Mongolia reach of the Yellow River, China. J. Mt. Sci..

[19] Lu XX, Ran LS, Liu S, Jiang T, Zhang SR, Wang JJ (2013). Sediment loads response to climate change: A preliminary study of eight large Chinese rivers. Int. J. Sediment. Res..

[20] Al-Wadaey A, Ziadat F (2014). A participatory GIS approach to identify critical land degradation areas and prioritize soil conservation for mountainous olive groves (case study). J. Mt. Sci..

[21] Han Y, Zheng F-L, Xu X-M (2017). Effects of rainfall regime and its character indices on soil loss at loessial hillslope with ephemeral gully. J. Mt. Sci..

[22] Sun, W. Temporal and Spatial Variation in Rainfall in Yanhe River Basin and Its Effect on Runoff and Sediment Variation. Unpublished master’s thesis/doctoral dissertation. Northwest Agriculture & Forestry University, Xianyang, China (2015).

[23] Li Y, Bai J, Zhou W, Song Y (2017). Effect of climatic factors on runoff and sediment variation in Bahe River Basin. Water Resour. Protect..

[24] Hou S, Hou K, Wang P, Chu W (2015). Analysis on impact of Liujiaxia Reservoir on variation of water and sediment. Water Resour. Hydropower Eng..

[25] Liu F, Chen S, Peng J, Chen G (2011). Temporal variability of water discharge and sediment load of the Yellow River into the sea during 1950–2008. J. Geogr. Sci..

[26] Zhao J, Yang Z, Govers G (2018). Soil and water conservation measures reduce soil and water losses in China but not down to background levels: evidence from erosion plot data. Geoderma.

[27] Zhang, H., Shi, J., Xin, C. & Gao, F. Variation trends analysis and its ecological impact of sediment discharge in the mainstream of the Yellow River. In *Proceedings on 2011 International Symposium of Water Resource Environment Protection.* May 20–22, Xi’an, China, 1124–1127, 10.1109/ISWREP.2011.5893212 (2011).

[28] Ran D, Jiao P, Yao W, Li X, Shang H (2015). Recent variation of runoff and sediment in response to the high intensity human activities in Dongchuan Basin of Jinghe River. J. Soil Water Conserv..

[29] Mu X, Wen Z, Wang F, Gao P (2013). Impacts of precipitation and human activities on streamflow and sediment load in the Huangfuchuan Watershed. Sci. Soil Water Conserv..

[30] Yan, M., Song, X., Xia, L. & Li, Y. Impact of LUCC and climate change on sediment load in the Yanwachuan Watershed on the losses plateau. In *Proceedings on International Conference of Management Engineering.* May 24–25, Shanghai, China, 174–178 (2014).

[31] Lin X, Shen G, Guo Y (2014). Analysis on taking water and diverting sediment conditions of the Yellow River Diversion Project in central Shanxi Province. Yellow River.

[32] Meshesha DT, Tsunekawa A, Tsubo M, Haregeweyn N (2012). Dynamics and hotspots of soil erosion and management scenarios of the Central Rift Valley of Ethiopia. Int. J. Sediment. Res..

[33] Adeogun AG, Sule BF, Salami AW (2016). Cost effectiveness of sediment management strategies for mitigation of sedimentation at Jebba Hydropower reservoir, Nigeria. J. King Saud Univ. Eng. Sci..

[34] Jiang G, Gao P, Mu X, Chai X (2015). Effect of conversion of farmland to forestland or grassland on the change in runoff and sediment in the upper reaches of Beiluo River. Res. Soil Water Conserv..

[35] Deng L, Shangguan Z-P, Li R (2012). Effects of the grain-for-green program on soil erosion in China. Int. J. Sediment. Res..

[36] Tao G, Gao Z, Hang H (2013). The effect on downstream reservoir runoff and sediment after running of Pubugou Hydropower Station. Hydropower New Energy.

[37] Liu N, Xie Y, Zhang C, Deng Z, Zhang C, Yao B (2014). Characteristics of water and sediment discharge in Lishui River and factor analysis. J. China Hydrol..

[38] Zhang J, Wang H, Zhang Y, Bi S (2012). Variation of sediment load at the major tributaries in the middle reaches of Yellow River and its impacts on the sediment flux to the sea. Mar. Geol. Quat. Geol..

[39] Zhao G, Mu X, Tian P, Wang F, Gao P (2012). The variation trend of streamflow and sediment flux in the middle reaches of Yellow River over the past 60 years and the influencing factors. Resour. Sci..

[40] Wang Y, Shi H (2011). Water and sediment diversion characteristics of various irrigation types and their influence on canal erosion and deposition in irrigation systems of Lower Yellow River. J. Sediment. Res..

[41] Zhang L (2014). The Relationship Between Vegetation and Runoff, Sediment in the Past 50 Years in Yanhe River Watershed.

[42] Yellow River Engineering Consulting (2010). Design of 2020 Runoff and sediment into Dongzhuang Reservoir.

[43] Yellow River Engineering Consulting (2017). Supplementary Feasibility Study Report on Dongzhuang Hydroproject on Jinghe River in Shaanxi Province.

[44] Yellow River Engineering Consulting (2018). Preliminary design report on Dongzhuang Hydroproject on Jinghe River in Shaanxi Province.

[45] Yellow River Basin Monitoring Center of Water-Soil Conservation and Eco-Environment (2013). Survey and Assessment Report on Soil and Water Conservation Practices in Upper and Middle Yellow River.

[46] Yao W, Xu J, Ran D (2011). Analysis and Assessment of Runoff and Sediment Variation Regime in Yellow River Basin.

[47] Liu X, Li X, Li Y, Tian Y, Dang S (2016). Causes of Sharp Decrease in Runoff and Sediment of Yellow River.

[48] Yellow River Conservancy Commission (2016). Study on Variation in Runoff and Sediment in Yellow River.

[49] Yellow River Conservancy Commission (2013). Comprehensive Planning of Yellow River Basin.

[50] Juez C, Schärer C, Jenny H, Schleiss AJ, Franca MJ (2019). Floodplain land cover and flow hydrodynamic control of overbank sedimentation in compound channel flows. Water Resour. Res..

